# Editorial: The impact of home and school environment on early literacy and mathematic skills

**DOI:** 10.3389/fpsyg.2023.1258391

**Published:** 2023-08-08

**Authors:** Paola Bonifacci, Valentina Tobia, Tomohiro Inoue, George Manolitsis

**Affiliations:** ^1^Department of Psychology, University of Bologna, Bologna, Italy; ^2^Faculty of Psychology, Vita-Salute San Raffaele University, Milan, Italy; ^3^Department of Psychology, The Chinese University of Hong Kong, Shatin, Hong Kong SAR, China; ^4^Department of Preschool Education, University of Crete, Rethymno, Greece

**Keywords:** home literacy, home numeracy, environmental factors, socioeconomic status, bilingualism

## Introduction

Early development of literacy and mathematics skills has been shown to be a cornerstone of children's later academic achievement (e.g., Shanahan and Lonigan, 2010; Watts et al., 2014). The roles of various environmental factors in the development of cognitive and academic skills have received increasing attention from researchers, practitioners, and parents. Current theoretical models such as the bioecological framework (Bronfenbrenner and Morris, 2006) and neuroconstructivism (Westermann et al., 2007) emphasize the dynamic reciprocal relationship between genetic, neurobiological, and environmental factors in child development. In light of this, a growing number of studies have examined the relationships between the home literacy and numeracy environment (HLE and HNE, respectively) and the development of these academic skills (Noble et al., 2019; for meta-analyses, see e.g., Daucourt et al., 2021). Several studies have also shed light on the influences of environmental factors outside the home, such as tutoring and schooling, where children primarily learn literacy and numeracy (e.g., Nag et al., 2019). Furthermore, the profound influences of more distal environmental factors such as family socioeconomic status (SES) and linguistic background (e.g., bilingualism) have been well documented (e.g., Sirin, 2005; Kim et al., 2019; Dong and Chow, 2022). Despite these collective efforts in the literature, the precise mechanisms driving the documented associations between factors inside and outside the child's rearing environment and literacy and numeracy development remain poorly understood. Thus, further research is warranted to unpack the complex developmental dynamics among these factors at different levels of analysis, including both distal and proximal factors.

In this Research Topic, we sought to address this issue and examine how environmental factors influence children's early literacy and numeracy, namely the roots of later academic achievement. Indeed, existing empirical studies have produced mixed findings that do not allow us to draw any definitive conclusions (e.g., Noble et al., 2019; Daucourt et al., 2021). For example, while most previous studies found a positive association between HNE and children's early numeracy (e.g., counting, number sense), the results varied widely in terms of the strength of the associations (see Daucourt et al., 2021). Studies on the role of shared book reading, an important aspect of HLE, have also reported mixed results (Noble et al., 2019). These heterogeneities in existing findings may be due, at least in part, to the involvement of other (possibly confounding) factors that may affect the home environment, early numeracy/literacy, or both. This may include family SES, ethnic and linguistic background (e.g., bilingualism), parental expectations and beliefs, parental attitudes toward literacy and numeracy, as well as parental practices (see Nag et al., 2019).

This Research Topic brings together a Research Topic of ten articles that explore the role of the home environment on literacy and numeracy skills from preschool to primary school in different contexts. The contributions depict a complex picture that underlies the multifaceted nature of home learning environment and of early literacy/numeracy, which includes many dimensions.

First, some studies focused on HLE and related children's skills. Tanji and Inoue reported differential effects of subdomains of HLE on reading skills in two different scripts of the Japanese writing system. In particular, the dimensions evaluated were parent teaching, shared book reading, and access to literacy resources. The results suggested that Japanese parents might adjust their involvement according to both their children's reading performance and social expectations for academic achievement.

Moving to school-age children and the analysis of writing skills, Su et al. examined the associations between the onset age of parental home teaching and the informal occasions of exposure to literacy outside the home (e.g., science center, art gallery). Their findings suggested a significant role of both dimensions. Also, in a longitudinal perspective, Bigozzi et al. showed that HLE predicted reading speed and writing accuracy from preschool to primary school, mediated by notational awareness. From a different perspective, Aram and Yashar evaluated parents' awareness of children's writing abilities. They suggested that parents' general knowledge and understanding of literacy development play a role in fostering their children's literacy skills.

Turning to the numeracy domain, Wei et al. revealed the role of home numeracy practices in a longitudinal study. Specifically, they showed a unidirectional relationship between parents' basic teaching activities (e.g., teaching counting) and subsequent basic number processing (i.e., digit comparison) and between advanced teaching activities (i.e., related to written numbers) and the following children's arithmetic skills. DePascale et al. add another piece to this picture by showing that home-based advanced math activities, literacy activities, and SES are all associated with strategy sophistication in solving numerical problems. Considering the importance of mathematics-related activities in the home environment, Tomasetto et al. offer promising evidence, showing how a non-intensive intervention program delivered by community pediatricians can improve parents' provision of these activities.

Finally, methodological issues were addressed. Eriksson et al. managed an original point related to estimating the number of books at home as a proxy for SES. By analyzing the data from a large international sample, they showed unsystematic errors in estimates of books, revealing an important risk for educational studies: The strength of the association between books at home and achievement may generally be underestimated, particularly in low-achieving countries and/or students. Similarly, Krousorati et al. noted the methodological limitations of the current literature, particularly about the conceptualization and measurement of the home learning environment. They proposed a home learning environment questionnaire that goes beyond the assessment of home learning activities and provides us with a wider range of information, including the quality of parent-child interactions (support, conflict, and inconsistent discipline).

To conclude, this Research Topic of articles highlights the importance of considering various aspects of children's learning environment, with the need for further validated tools that embrace the different dimensions and extend current theoretical models of HLE and HNE. Future development should also include the evaluation of other environmental variables, such as the school domain, and combine them into integrated models of how contextual variables dynamically impact children's early literacy and numeracy development.

## Author contributions

All authors listed have made a substantial, direct, and intellectual contribution to the work and approved it for publication.

## References

[B1] Bronfenbrenner U. Morris P. A. (2006). “The bioecological model of human development,” in Handbook of Child Psychology: Theoretical Models of Human Development, eds R. M. Lerner and W. Damon (London: John Wiley and Sons, Inc), 793–828.

[B2] Daucourt M. C. Napoli A. R. Quinn J. M. Wood S. G. Hart S. A. (2021). The home math environment and math achievement: a meta-analysis. Psychol. Bullet. 147, 565–596. 10.1037/bul0000330 34843299 PMC8634776

[B3] Dong Y. Chow B. W. Y. (2022). Home literacy environment and English as a second language acquisition: a meta-analysis. Lang. Learn. Dev. 18, 485–499. 10.1080/15475441.2021.2003197

[B4] Kim S. Cho H. Kim L. Y. (2019). Socioeconomic status and academic outcomes in developing countries: a meta-analysis. Rev. Educ. Res. 89, 875–916. 10.3102/0034654319877155

[B5] Nag S. Vagh S. B. Dulay K. M. Snowling M. J. (2019). Home language, school language and children's literacy attainments: a systematic review of evidence from low- and middle-income countries. Rev. Educ. 7, 91–150. 10.1002/rev3.3132

[B6] Noble C. Sala G. Peter M. Lingwood J. Rowland C. Gobet F.. (2019). The impact of shared book reading on children's language skills_ A meta-analysis. Educ. Res. Rev. 28, 100290. 10.1016/j.edurev.2019.100290

[B7] Shanahan T. Lonigan C. J. (2010). The national early literacy panel. Educ. Res. 39, 279–285. 10.3102/0013189X10369172 PMC3268038 22294803

[B8] Sirin S. R. (2005). Socioeconomic status and academic achievement: a meta-analytic review of research. Rev. Educ. Res. 75, 417–453. 10.3102/00346543075003417

[B9] Watts T. W. Duncan G. J. Siegler R. S. Davis-Kean P. E. (2014). What's past is prologue: relations between early mathematics knowledge and high school achievement. Educ. Res. 43, 352–360. 10.3102/0013189X14553660 26806961 PMC4719158

[B10] Westermann G. Mareschal D. Johnson M. H. Sirois S. Spratling M. W. Thomas M. S. C.. (2007). Neuroconstructivism. Dev. Sci. 10, 75–83. 10.1111/j.1467-7687.2007.00567.x 17181703

